# (5,7-Dimethyl-2-oxo-2*H*-chromen-4-yl)methyl pyrrolidine-1-carbodithio­ate

**DOI:** 10.1107/S1600536812018004

**Published:** 2012-05-02

**Authors:** N. M. Mahabaleshwaraiah, K. Mahesh Kumar, O. Kotresh, Waleed Fadl Ali Al-eryani, H. C. Devarajegowda

**Affiliations:** a Department of Chemistry, Karnatak Science College, Dharwad 580 001, Karnataka, India; b Department of Physics, Yuvaraja’s College (Constituent College), University of Mysore, Mysore 570 005, Karnataka, India

## Abstract

In the title compound, C_17_H_19_NO_2_S_2_, the 2*H*-chromene ring system is almost planar, with a maximum deviation of 0.044 (2) Å, and the pyrrolidine ring adopts an envelope conformation. The dihedral angle between the 2*H*-chromene system and the planar part of the pyrrolidine ring is 83.65 (8)°. A weak intra­molecular C—H⋯S hydrogen bond occurs. The crystal structure features C—H⋯O hydrogen bonds and π–π inter­actions, with a centroid–centroid distance of 3.5728 (16) Å.

## Related literature

For biological properties of coumarins, see: Adavi *et al.* (2004 ▶); Laurin *et al.* (1999 ▶); Kulkarni *et al.* (2006 ▶). For related structures, see: Kumar *et al.* (2012 ▶); Kant *et al.* (2012 ▶). For synthetic details, see: Shastri *et al.* (2004 ▶); Vasilliev & Polackov (2000 ▶).
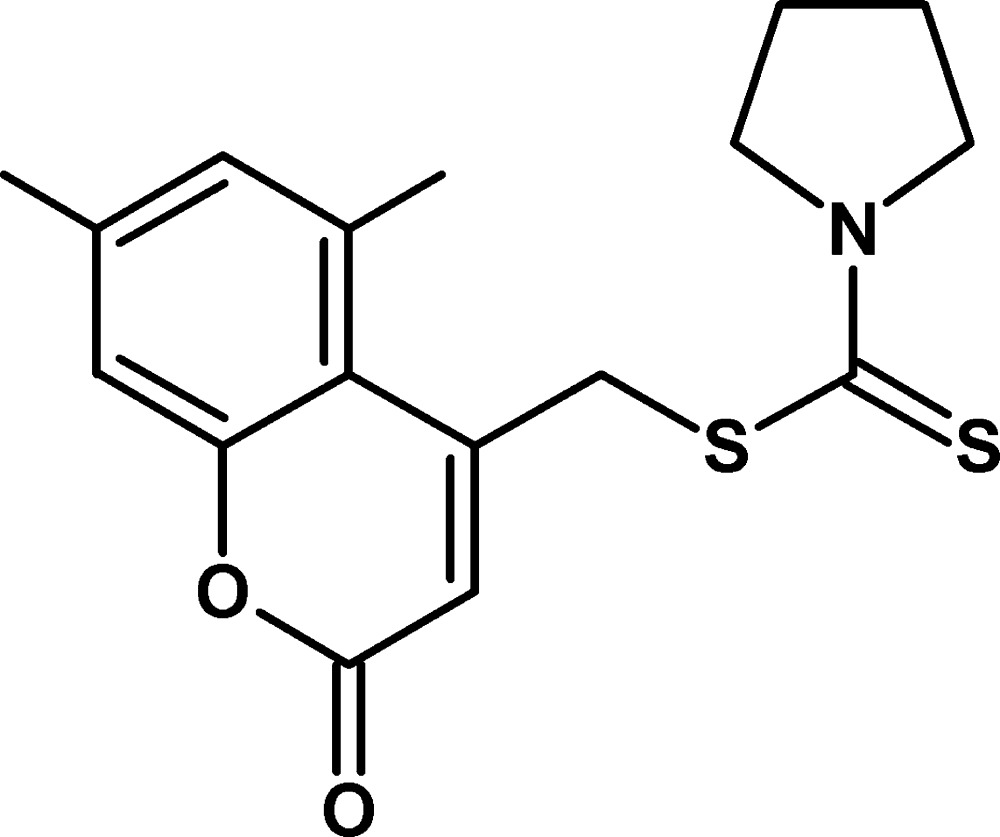


## Experimental

### 

#### Crystal data

C_17_H_19_NO_2_S_2_
*M* *_r_* = 333.45
Monoclinic, 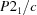

*a* = 13.7023 (4) Å
*b* = 15.9082 (4) Å
*c* = 7.5511 (2) Å
β = 103.358 (1)°
*V* = 1601.45 (7) Å^3^
*Z* = 4
Mo *K*α radiation
μ = 0.34 mm^−1^
*T* = 293 K
0.24 × 0.20 × 0.12 mm

#### Data collection

Bruker SMART CCD area-detector diffractometer
Absorption correction: ψ scan (*SADABS*; Sheldrick, 2007 ▶) *T*_min_ = 0.770, *T*_max_ = 1.000
12677 measured reflections
2805 independent reflections
2410 reflections with *I* > 2σ(*I*)
*R*_int_ = 0.024

#### Refinement

*R*[*F* ^2^ > 2σ(*F* ^2^)] = 0.035
*wR*(*F* ^2^) = 0.099
*S* = 1.07
2805 reflections
201 parameters
H-atom parameters constrained
Δρ_max_ = 0.33 e Å^−3^
Δρ_min_ = −0.26 e Å^−3^

### 

Data collection: *SMART* (Bruker, 2001 ▶); cell refinement: *SAINT* (Bruker, 2001 ▶); data reduction: *SAINT*; program(s) used to solve structure: *SHELXS97* (Sheldrick, 2008 ▶); program(s) used to refine structure: *SHELXL97* (Sheldrick, 2008 ▶); molecular graphics: *ORTEP-3* (Farrugia, 1997 ▶); software used to prepare material for publication: *SHELXL97*.

## Supplementary Material

Crystal structure: contains datablock(s) I, global. DOI: 10.1107/S1600536812018004/wn2472sup1.cif

Structure factors: contains datablock(s) I. DOI: 10.1107/S1600536812018004/wn2472Isup2.hkl

Supplementary material file. DOI: 10.1107/S1600536812018004/wn2472Isup3.cml

Additional supplementary materials:  crystallographic information; 3D view; checkCIF report

## Figures and Tables

**Table 1:** Hydrogen-bond geometry (Å, °)

*D*—H⋯*A*	*D*—H	H⋯*A*	*D*⋯*A*	*D*—H⋯*A*
C11—H11*B*⋯S2	0.97	2.51	3.172 (2)	126
C18—H18⋯O4^i^	0.93	2.52	3.411 (3)	161
C22—H22*C*⋯S1	0.96	2.81	3.564 (2)	137

Symmetry code: (i) 
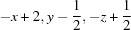
.

**Fig. 1.**
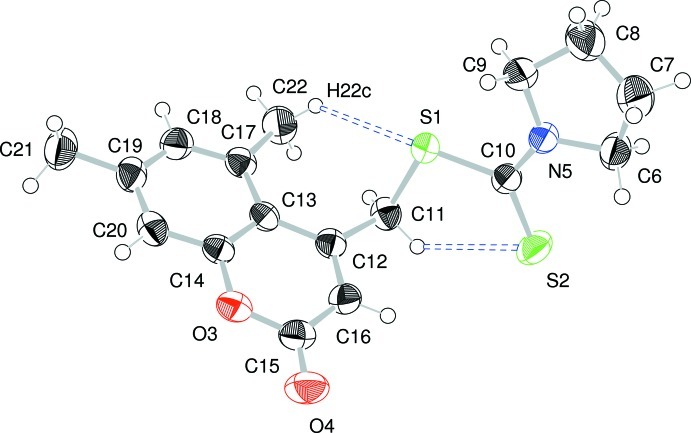
The molecular structure of the title compound. Displacement ellipsoids are drawn at the 50% probability level. Hydrogen atoms are shown as spheres of arbitrary radius. Dashed lines indicate intramolecular hydrogen bonds.

**Fig. 2.**
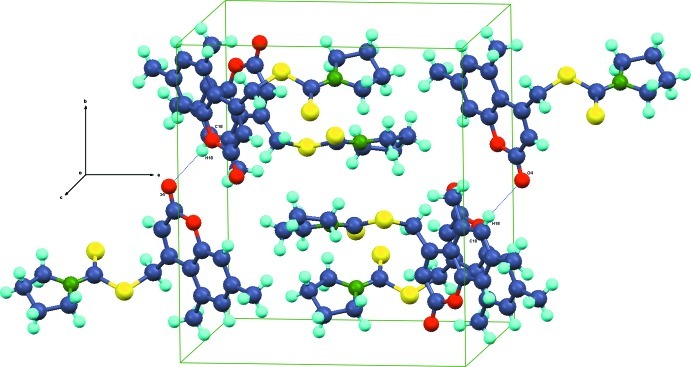
The packing of molecules in the title structure, viewed down the c-axis. Dashed lines indicate intermolecular hydrogen bonds.

## supplementary crystallographic information

### Comment

Coumarins are a class of naturally occurring lactones. A number of coumarins have been isolated in recent years, mainly from plant sources and extracts of these have been employed as traditional medicines in different areas of the world. Coumarin derivatives with various thio substituents at the C-4 position have revealed potential as antibacterial (Adavi *et al.*, 2004), DNA gyrase studies (Laurin *et al.*, 1999) and anticancer activity (Kularni *et al.*, 2006).

In our present work, we have been able to link a dithiocarbamate group at the C-4 methylene carbon and it was deemed of considerable interest to study the effect of this group on the total solid state conformation of the molecule. The synthesized compound was screened for antimicrobial, antidiabetic, DNA binding and DNA cleavage studies.In continuation of our interest in the crystal structures of coumarin derivatives (Kumar *et al.*, 2012; Kant *et al.*, 2012), we report here the crystal structure of the title compound.

The asymmetric unit of (5,7-dimethyl-2-oxo-2*H*-chromen-4-yl)methyl pyrrolidine-1-carbodithioate is shown in Fig. 1. The 2*H*-chromene ring system (O3/C12–C20) is essentially planar, with a maximum deviation of 0.044 (2)Å for atom C15.The pyrrolidine ring adopts an envelope conformation with C8 as the flap atom. The dihedral angle between the 2*H*-chromene ring system (O3/C12–C20) and the planar part of the pyrrolidine ring (N5, C6, C7, C9) is 83.65 (8)°.

In the crystal structure, (Fig. 2), the molecules are connected *via* weak intramolecular C11—H11B···S2 and C22—H22C···S1 and intermolecular C—H···O hydrogen bonds (Table 1).Furthermore, the crystal structure features a π-π interaction,with a centroid *Cg*2 (O3/C12–C16) to centroid *Cg*3 (C13/C14/C17–C20) distance of 3.5728 (16) Å.

### Experimental

All the chemicals used were of analytical reagent grade and were used directly without further purification.4-Bromomethyl coumarin required for the synthesis of the target molecule was synthesized according to an already reported procedure involving Pechmann cyclization of phenols with 4-bromoethyl acetoacetate (Shastri *et al.*, 2004) and sodium pyrrolidine-1-carbodithioate was synthesized according to the reported procedure (Vasilliev & Polackov, 2000). A mixture of 2.67 g (0.01 mol) of 5,7-dimethyl-4-bromomethyl coumarin and1.69 g (0.01 mol) of sodium pyrrolidine-1-carbodithioate in 30 ml dry alcohol was stirred for 24 h at room temperature (the reaction was monitored by TLC). The solvent was evaporated and the resulting solid was extracted twice with a dichloromethane-H_2_O mixture.The organic layer was dried over anhydrous CaCl_2_ and evaporation of the organic solvent gave the title compound.The compound was recrystallized from an ethanol-chloroform mixture. Colour: colourless. Yield 88%, m.p.443 K.

### Refinement

All H atoms were positioned geometrically, with C—H = 0.93 Å for aromatic H, C—H = 0.97 Å for methylene H and C—H = 0.96 Å for methyl H,and refined using a riding model with *U*_iso_(H) = 1.5*U*_eq_(C) for methyl H and *U*_iso_(H) = 1.2*U*_eq_(C) for all other H.

### Crystal data

**Table:**

C_17_H_19_NO_2_S_2_	*F*(000) = 704
*M**_r_* = 333.45	*D*_x_ = 1.383 Mg m^−^^3^
Monoclinic, *P*2_1_/*c*	Melting point: 443 K
Hall symbol: -P 2ybc	Mo *K*α radiation, λ = 0.71073 Å
*a* = 13.7023 (4) Å	Cell parameters from 2805 reflections
*b* = 15.9082 (4) Å	θ = 1.5–25.0°
*c* = 7.5511 (2) Å	µ = 0.34 mm^−^^1^
β = 103.358 (1)°	*T* = 293 K
*V* = 1601.45 (7) Å^3^	Plate, colourless
*Z* = 4	0.24 × 0.20 × 0.12 mm

### Data collection

**Table:**

Bruker SMART CCD area-detector diffractometer	2805 independent reflections
Radiation source: fine-focus sealed tube	2410 reflections with *I* > 2σ(*I*)
Graphite monochromator	*R*_int_ = 0.024
ω and φ scans	θ_max_ = 25.0°, θ_min_ = 1.5°
Absorption correction: ψ scan (*SADABS*; Sheldrick, 2007)	*h* = −12→16
*T*_min_ = 0.770, *T*_max_ = 1.000	*k* = −17→18
12677 measured reflections	*l* = −8→8

### Refinement

**Table:**

Refinement on *F*^2^	Primary atom site location: structure-invariant direct methods
Least-squares matrix: full	Secondary atom site location: difference Fourier map
*R*[*F*^2^ > 2σ(*F*^2^)] = 0.035	Hydrogen site location: inferred from neighbouring sites
*wR*(*F*^2^) = 0.099	H-atom parameters constrained
*S* = 1.07	*w* = 1/[σ^2^(*F*_o_^2^) + (0.052*P*)^2^ + 0.452*P*] where *P* = (*F*_o_^2^ + 2*F*_c_^2^)/3
2805 reflections	(Δ/σ)_max_ = 0.001
201 parameters	Δρ_max_ = 0.33 e Å^−^^3^
0 restraints	Δρ_min_ = −0.26 e Å^−^^3^

### Special details

**Table:**

Experimental. IR (KBr): 686 cm^-1^ (C—S), 1226.8 cm^-1^ (C=S), 1000 cm^-1^ (C—O), 859 cm^-1^ (C—N),1153 cm^-1^ (C—O—C), 1719.7 cm^-1^ (C=O). GCMS: m/e: 333. 1H NMR (400 MHz, DMSO.D6, \?, p.p.m.): 1.91 (m,2*H*, C_11_), 2.02 (m,2*H*, C_1_), 2.49 (s,3*H*, C_17_), 2.74 (s,3*H*, C_10_), 3.66 (t,2*H*, C_2_), 4.8 (d,2*H*, C_4_), 6.5 (s,1*H*, C_14_), 7.03 (s,1*H*, C_15_), 7.10 (s,1*H*, C_8_). Elemental analysis: C, 61.20; H, 5.70; N, 4.16; O, 9.57; S, 19.19.
Geometry. All s.u.'s (except the s.u. in the dihedral angle between two l.s. planes) are estimated using the full covariance matrix. The cell s.u.'s are taken into account individually in the estimation of s.u.'s in distances, angles and torsion angles; correlations between s.u.'s in cell parameters are only used when they are defined by crystal symmetry. An approximate (isotropic) treatment of cell s.u.'s is used for estimating s.u.'s involving l.s. planes.
Refinement. Refinement of *F*^2^ against ALL reflections. The weighted *R*-factor *wR* and goodness of fit *S* are based on *F*^2^, conventional *R*-factors *R* are based on *F*, with *F* set to zero for negative *F*^2^. The threshold expression of *F*^2^ > 2σ(*F*^2^) is used only for calculating *R*-factors(gt) *etc*. and is not relevant to the choice of reflections for refinement. *R*-factors based on *F*^2^ are statistically about twice as large as those based on *F*, and *R*- factors based on ALL data will be even larger.

### Fractional atomic coordinates and isotropic or equivalent isotropic displacement parameters (Å^2^)

**Table:**

	*x*	*y*	*z*	*U*_iso_*/*U*_eq_	
S1	0.65286 (3)	0.09638 (3)	0.11104 (6)	0.04453 (16)	
S2	0.49960 (4)	0.17744 (4)	−0.20084 (7)	0.05921 (19)	
O3	0.92005 (10)	0.33298 (8)	0.23621 (19)	0.0473 (3)	
O4	0.81069 (13)	0.42553 (9)	0.0928 (2)	0.0666 (4)	
N5	0.46456 (11)	0.11498 (9)	0.1028 (2)	0.0407 (4)	
C6	0.35762 (14)	0.13604 (14)	0.0441 (3)	0.0529 (5)	
H6A	0.3243	0.1002	−0.0556	0.063*	
H6B	0.3485	0.1943	0.0058	0.063*	
C7	0.31788 (18)	0.12064 (18)	0.2114 (4)	0.0707 (7)	
H7A	0.3155	0.1728	0.2766	0.085*	
H7B	0.2508	0.0973	0.1779	0.085*	
C8	0.38742 (17)	0.06053 (18)	0.3262 (3)	0.0705 (7)	
H8A	0.3658	0.0031	0.2966	0.085*	
H8B	0.3904	0.0703	0.4541	0.085*	
C9	0.48806 (15)	0.07603 (13)	0.2847 (3)	0.0501 (5)	
H9A	0.5282	0.1135	0.3740	0.060*	
H9B	0.5243	0.0237	0.2837	0.060*	
C10	0.52973 (13)	0.13150 (11)	0.0029 (2)	0.0387 (4)	
C11	0.72925 (14)	0.13642 (12)	−0.0367 (3)	0.0451 (4)	
H11A	0.7657	0.0904	−0.0757	0.054*	
H11B	0.6862	0.1612	−0.1443	0.054*	
C12	0.80249 (13)	0.20148 (11)	0.0595 (2)	0.0393 (4)	
C13	0.89901 (13)	0.18216 (11)	0.1824 (2)	0.0373 (4)	
C14	0.95351 (13)	0.25130 (11)	0.2690 (2)	0.0391 (4)	
C15	0.83239 (15)	0.35208 (12)	0.1143 (3)	0.0477 (5)	
C16	0.77386 (14)	0.28199 (12)	0.0298 (3)	0.0455 (4)	
H16	0.7126	0.2929	−0.0501	0.055*	
C17	0.94441 (14)	0.10202 (11)	0.2242 (3)	0.0424 (4)	
C18	1.03608 (15)	0.09721 (13)	0.3483 (3)	0.0490 (5)	
H18	1.0654	0.0446	0.3742	0.059*	
C19	1.08660 (14)	0.16670 (13)	0.4363 (3)	0.0473 (5)	
C20	1.04425 (14)	0.24450 (13)	0.3941 (3)	0.0459 (4)	
H20	1.0765	0.2924	0.4495	0.055*	
C21	1.18405 (16)	0.15628 (17)	0.5764 (3)	0.0660 (6)	
H21A	1.2304	0.1991	0.5594	0.099*	
H21B	1.2119	0.1019	0.5628	0.099*	
H21C	1.1719	0.1611	0.6962	0.099*	
C22	0.90076 (17)	0.02021 (13)	0.1408 (3)	0.0652 (6)	
H22A	0.9452	−0.0251	0.1893	0.098*	
H22B	0.8926	0.0226	0.0112	0.098*	
H22C	0.8367	0.0111	0.1688	0.098*	

### Atomic displacement parameters (Å^2^)

**Table:**

	*U*^11^	*U*^22^	*U*^33^	*U*^12^	*U*^13^	*U*^23^
S1	0.0365 (3)	0.0496 (3)	0.0446 (3)	−0.0019 (2)	0.00356 (19)	0.0083 (2)
S2	0.0570 (3)	0.0720 (4)	0.0455 (3)	0.0119 (3)	0.0052 (2)	0.0175 (2)
O3	0.0530 (8)	0.0355 (7)	0.0542 (8)	−0.0048 (6)	0.0141 (6)	0.0003 (6)
O4	0.0780 (11)	0.0392 (8)	0.0832 (11)	0.0045 (7)	0.0199 (9)	0.0115 (7)
N5	0.0378 (8)	0.0393 (8)	0.0424 (8)	0.0008 (6)	0.0040 (6)	0.0018 (6)
C6	0.0391 (10)	0.0573 (13)	0.0595 (12)	0.0071 (9)	0.0059 (9)	0.0022 (10)
C7	0.0510 (13)	0.0842 (18)	0.0813 (17)	0.0068 (12)	0.0243 (12)	0.0095 (14)
C8	0.0605 (14)	0.0930 (18)	0.0605 (14)	−0.0091 (13)	0.0189 (11)	0.0084 (13)
C9	0.0501 (11)	0.0541 (12)	0.0444 (11)	−0.0031 (9)	0.0072 (8)	0.0071 (9)
C10	0.0410 (10)	0.0315 (9)	0.0402 (9)	−0.0008 (7)	0.0023 (7)	−0.0030 (7)
C11	0.0425 (10)	0.0512 (12)	0.0418 (10)	−0.0034 (8)	0.0104 (8)	−0.0031 (8)
C12	0.0392 (9)	0.0450 (10)	0.0366 (9)	−0.0011 (8)	0.0146 (7)	0.0016 (7)
C13	0.0358 (9)	0.0412 (10)	0.0383 (9)	−0.0022 (7)	0.0157 (7)	0.0002 (7)
C14	0.0409 (9)	0.0394 (10)	0.0408 (9)	−0.0022 (8)	0.0172 (7)	0.0008 (7)
C15	0.0536 (12)	0.0427 (11)	0.0507 (11)	0.0021 (9)	0.0197 (9)	0.0071 (8)
C16	0.0443 (10)	0.0477 (11)	0.0451 (10)	0.0020 (9)	0.0114 (8)	0.0080 (8)
C17	0.0402 (10)	0.0392 (10)	0.0512 (11)	0.0009 (8)	0.0177 (8)	0.0008 (8)
C18	0.0436 (11)	0.0486 (12)	0.0573 (12)	0.0076 (9)	0.0166 (9)	0.0074 (9)
C19	0.0354 (10)	0.0655 (13)	0.0432 (10)	0.0005 (9)	0.0139 (8)	0.0035 (9)
C20	0.0410 (10)	0.0539 (12)	0.0445 (10)	−0.0091 (9)	0.0135 (8)	−0.0039 (8)
C21	0.0441 (12)	0.0919 (18)	0.0591 (14)	0.0032 (12)	0.0062 (10)	0.0067 (12)
C22	0.0555 (13)	0.0413 (12)	0.0949 (17)	0.0030 (10)	0.0093 (12)	−0.0052 (11)

### Geometric parameters (Å, º)

**Table:**

S1—C10	1.7858 (18)	C11—H11B	0.9700
S1—C11	1.8128 (19)	C12—C16	1.343 (3)
S2—C10	1.6667 (18)	C12—C13	1.462 (2)
O3—C15	1.368 (2)	C13—C14	1.403 (2)
O3—C14	1.381 (2)	C13—C17	1.422 (2)
O4—C15	1.207 (2)	C14—C20	1.381 (3)
N5—C10	1.322 (2)	C15—C16	1.434 (3)
N5—C6	1.468 (2)	C16—H16	0.9300
N5—C9	1.473 (2)	C17—C18	1.385 (3)
C6—C7	1.507 (3)	C17—C22	1.508 (3)
C6—H6A	0.9700	C18—C19	1.389 (3)
C6—H6B	0.9700	C18—H18	0.9300
C7—C8	1.481 (3)	C19—C20	1.373 (3)
C7—H7A	0.9700	C19—C21	1.509 (3)
C7—H7B	0.9700	C20—H20	0.9300
C8—C9	1.503 (3)	C21—H21A	0.9600
C8—H8A	0.9700	C21—H21B	0.9600
C8—H8B	0.9700	C21—H21C	0.9600
C9—H9A	0.9700	C22—H22A	0.9600
C9—H9B	0.9700	C22—H22B	0.9600
C11—C12	1.506 (3)	C22—H22C	0.9600
C11—H11A	0.9700		
			
C10—S1—C11	103.15 (9)	C16—C12—C11	116.00 (17)
C15—O3—C14	122.22 (14)	C13—C12—C11	124.46 (16)
C10—N5—C6	122.74 (15)	C14—C13—C17	116.15 (16)
C10—N5—C9	125.80 (15)	C14—C13—C12	115.91 (16)
C6—N5—C9	111.45 (15)	C17—C13—C12	127.94 (16)
N5—C6—C7	103.79 (17)	C20—C14—O3	113.91 (16)
N5—C6—H6A	111.0	C20—C14—C13	123.74 (17)
C7—C6—H6A	111.0	O3—C14—C13	122.34 (16)
N5—C6—H6B	111.0	O4—C15—O3	117.15 (19)
C7—C6—H6B	111.0	O4—C15—C16	126.7 (2)
H6A—C6—H6B	109.0	O3—C15—C16	116.13 (16)
C8—C7—C6	106.72 (19)	C12—C16—C15	123.74 (18)
C8—C7—H7A	110.4	C12—C16—H16	118.1
C6—C7—H7A	110.4	C15—C16—H16	118.1
C8—C7—H7B	110.4	C18—C17—C13	118.81 (17)
C6—C7—H7B	110.4	C18—C17—C22	116.43 (17)
H7A—C7—H7B	108.6	C13—C17—C22	124.75 (17)
C7—C8—C9	105.61 (19)	C17—C18—C19	123.60 (18)
C7—C8—H8A	110.6	C17—C18—H18	118.2
C9—C8—H8A	110.6	C19—C18—H18	118.2
C7—C8—H8B	110.6	C20—C19—C18	117.98 (17)
C9—C8—H8B	110.6	C20—C19—C21	121.32 (19)
H8A—C8—H8B	108.7	C18—C19—C21	120.68 (19)
N5—C9—C8	104.47 (16)	C19—C20—C14	119.65 (18)
N5—C9—H9A	110.9	C19—C20—H20	120.2
C8—C9—H9A	110.9	C14—C20—H20	120.2
N5—C9—H9B	110.9	C19—C21—H21A	109.5
C8—C9—H9B	110.9	C19—C21—H21B	109.5
H9A—C9—H9B	108.9	H21A—C21—H21B	109.5
N5—C10—S2	123.94 (13)	C19—C21—H21C	109.5
N5—C10—S1	111.60 (13)	H21A—C21—H21C	109.5
S2—C10—S1	124.45 (11)	H21B—C21—H21C	109.5
C12—C11—S1	111.07 (12)	C17—C22—H22A	109.5
C12—C11—H11A	109.4	C17—C22—H22B	109.5
S1—C11—H11A	109.4	H22A—C22—H22B	109.5
C12—C11—H11B	109.4	C17—C22—H22C	109.5
S1—C11—H11B	109.4	H22A—C22—H22C	109.5
H11A—C11—H11B	108.0	H22B—C22—H22C	109.5
C16—C12—C13	119.52 (17)		

### Hydrogen-bond geometry (Å, º)

**Table:**

*D*—H···*A*	*D*—H	H···*A*	*D*···*A*	*D*—H···*A*
C11—H11*B*···S2	0.97	2.51	3.172 (2)	126
C18—H18···O4^i^	0.93	2.52	3.411 (3)	161
C22—H22*C*···S1	0.96	2.81	3.564 (2)	137

Symmetry code: (i) −*x*+2, *y*−1/2, −*z*+1/2.
